# Down‐regulated lncRNA SBF2‐AS1 in M2 macrophage‐derived exosomes elevates miR‐122‐5p to restrict XIAP, thereby limiting pancreatic cancer development

**DOI:** 10.1111/jcmm.15125

**Published:** 2020-04-16

**Authors:** Zi Yin, Yu Zhou, Tingting Ma, Sheng Chen, Ning Shi, Yiping Zou, Baohua Hou, Chuanzhao Zhang

**Affiliations:** ^1^ General Surgery Department Guangdong Provincial People's Hospital Guangdong Academy of Medical Sciences Guangzhou China; ^2^ Obstetrics and Gynecology Department Sun Yat‐Sen Memorial Hospital of Sun Yat‐sen University Guangzhou China

**Keywords:** exosomes, LncRNA SBF2‐AS1, macrophages, MicroRNA‐122‐5p, pancreatic cancer, X‐linked inhibitor of apoptosis protein

## Abstract

Evidence has indicated that M2 macrophages promote the progression of cancers, but few focus on the ability of M2 macrophage‐derived exosomes in pancreatic cancer (PC). This study aims to explore how M2 macrophages affect malignant phenotypes of PC through regulating long non‐coding RNA SET‐binding factor 2 antisense RNA 1 (lncRNA SBF2‐AS1)/microRNA‐122‐5p (miR‐122‐5p)/X‐linked inhibitor of apoptosis protein (XIAP) axis. THP‐1 cells were transformed into M1 macrophages by lipopolysaccharide and interferon‐γ treatment, and into M2 macrophages after interleukin‐4 treatment. The PANC‐1 PC cell line with the largest lncRNA SBF2‐AS1 expression was selected, and M2 macrophage‐derived exosomes were isolated and identified. A number of assays were applied for the examination of lncRNA SBF2‐AS1 expression, PC cell biological functions and subcellular localization of lncRNA SBF2‐AS1. XIAP expression was detected, along with the interaction among lncRNA SBF2‐AS1, miR‐122‐5p and XIAP. M2 macrophage exosomal lncRNA SBF2‐AS1 expression's effects on the tumorigenic ability of PANC‐1 cells in nude mice were also investigated. M2 macrophage‐derived exosomes promoted progression of PC cells. Overexpressed lncRNA SBF2‐AS1 promoted progression of PC cells. LncRNA SBF2‐AS1 was found to act as a competing endogenous RNA to repress miR‐122‐5p and up‐regulate XIAP. Constrained lncRNA SBF2‐AS1 in M2 macrophage‐derived exosomes contributed to restraining tumorigenic ability of PC cells. Collectively, our study reveals that constrained lncRNA SBF2‐AS1 in M2 macrophage‐derived exosomes increases miR‐122‐5p expression to restrain XIAP expression, which further inhibits PC progression.

## 1 INTRODUCTION

Pancreatic cancer (PC) is a major cause accounting for the cancer mortality in developed countries, and it is mainly comprised of adenocarcinoma (85%) and pancreatic endocrine tumours (under 5%).^1^ There are a multitude of risk factors in PC, including smoking, obesity, alcoholism, age, genetic factors and pre‐existing chronic pancreatitis.^2^ PC is chiefly manifested by anorexia, asthenia abdominal pain and weight loss, but there is often deferred diagnosis as a result of no particular symptoms.^3^ Moreover, little improvement has been made in the prevention and treatment of advanced PC patients.^4^ As PC causes great threats to human lives, this study aimed to make new explorations of therapies for PC treatment on the basis of previous studies.

Macrophages are considered as differentiated cells of the mononuclear phagocytic lineage which feature special phenotypic characteristics and particular marker expression, and have been reported to have positive effects on the tumorigenesis and tumour prognosis.^5^ In recent years, alternatively activated (M2) macrophages have been reported to play a critical part in gastric cancer development.^6^ Macrophage‐derived exosomes are capable of modulating drug resistance in pancreatic adenocarcinoma.^7^ Long non‐coding RNAs (lncRNAs) are a kind of ncRNAs that possess over 200 nucleotides and can function in gene expression and some tumours’ development.^8^ As a newly discovered lncRNA, SET‐binding factor 2 antisense RNA 1 (SBF2‐AS1) has been found to be involved in non–small‐cell lung cancer and results in unfavourable prognosis in patients.^9^ By functioning as a competitive endogenous RNA, lncRNA SBF2‐AS1 is found to elevate twinfilin‐1 (TWF1) to sponge miR‐142‐3p and participate in gemcitabine resistance in PC.^10^ Lon*g *et al^11^ have demonstrated that SBF2 up‐regulation can substantially depress cell proliferation and promote apoptosis in PC possibly through restriction of transforming growth factor β/SMAD signalling pathway. There is a study showing that in cervical cancer, lncRNA SBF2‐AS1 regulates microRNA (miRNA)‐361‐5p/forkhead box M1 (FOXM1) axis to contribute to tumour progression.^12^ MiRNAs have been proposed to correlate with cancer development.^13^ There is evidence indicating that miR‐122 is down‐regulated in PC.^14^ Besides that, lower miR‐122‐5p expression is demonstrated in PC tissues.^15^ A study has showed that X‐linked inhibitor of apoptosis protein (XIAP) is a direct target of miR‐130,^16^ and it is a cytosolic suppressor of caspases 3, 7 and 9.^17^ Young Kim et al^18^ have revealed that XIAP silencing is able to increase PC cell apoptosis stimulated by tumour necrosis factor‐related apoptosis‐inducing ligand. Nevertheless, few studies have paid attention to the role of M2 macrophage‐derived exosomes in PC. Hence, this study is meant to discuss how M2 macrophages affect malignant phenotypes of PC through lncRNA SBF2‐AS1/miR‐122‐5p/XIAP axis.

## 2 MATERIALS AND METHODS

### 2.1 Cell culture

PC cell lines (PANC‐1, BxPC‐3, SW1990, Capan‐2) and human mononuclear macrophage cell line (THP‐1) were purchased from the cell bank of American Type Culture Collection (https://www.atcc.org/). The complete medium used was centrifuged at 100 000 *g* to eliminate the exosomes.^19^ PC cells were cultured in Dulbecco's modified Eagle Medium (31600‐034, Hyclone Laboratories Inc) containing 10% foetal bovine serum (FBS) (10099141, Gibco).^20^, ^21^ THP‐1 cells were cultivated in Roswell Park Memorial Institute (RPMI)‐1640 medium 22 with 10% FBS. All cells were fostered at 37°C with 5% CO_2_. After the cell confluence reached 90%, the cells were detached and passaged at the ratio of 1:3‐4.^23^ All the cell lines used were free of mycoplasma contamination which was verified by short tandem repeat.^23^ THP‐1 cells were treated with 100 ng/mL polarized 12‐myristate 13‐acetate (P8139, from Sigma) for 24 hours for differentiation into macrophages, 100 ng/mL lipopolysaccharide (LPS, 8630, Sigma) and 20 ng/mL interferon‐γ (IFN‐γ, 285‐IF, R&D) for 24 hours for differentiation into M1 macrophages, and 20 ng/mL interleukin‐4 (IL‐4) (AF‐200‐04‐5, PeproTech) for 72 hours for differentiation into M2 macrophages.^7^

### 2.2 Exosome isolation and identification

Exosomes were isolated by ultracentrifugation. The cell culture supernatant of M2 macrophages was centrifuged at 500 *g* for 10 minutes to remove the precipitates, and the supernatant was centrifuged at 2000 *g* for 10 minutes at 4°C to remove cell debris. The obtained supernatant was filtered through a 0.22‐μm filter and centrifuged at 100 000 *g* for 4 hours in an ultracentrifuge tube. The precipitates were resuspended in phosphate‐buffered saline (PBS) and centrifuged at 100 000 *g* for 70 min. The obtained precipitates were exosomes.^24^

Exosome morphology was detected by a transmission electron microscope (TEM). The obtained exosomes (50 μL) were dropped on a 200‐mesh copper mesh and incubated at room temperature for 5 minutes. Then, the exosomes were stained with 1% phosphotungstic acid for 1 minute and rinsed 1 time or 2 times with distilled water. After drying, the copper mesh was observed with a TEM‐1400 plus at 80 kV. Nanoparticle tracking analysis: The exosomes were diluted to 3 × 10^7 ^− 5 × 10^7^ particles/mL with PBS, detected by a ZetaView PMX 110 instrument and analysed by ZetaView 8. 04. 02 SP2.

Exosome surface markers were identified by Western blot analysis, and the exosome protein content was measured by bicinchoninic acid (BCA) kit (23227, Thermo Fisher Scientific) after the concentration of the exosome suspension. Sodium dodecyl sulphate‐polyacrylamide gel electrophoresis was prepared, followed by protein denaturation and electrophoresis. Then, the exosomes were transferred to the membrane, and the expression of exosome‐specific marker protein TSG101, CD63, CD81 and GRP94 (1:1000, Abcam Inc.) was examined.

### 2.3 Exosome uptake

The exosomes dissolved in PBS were supplemented to 0.3 mL Diluent C for dilution and then stained with 4 μL PKH67 staining solution (Sigma) and incubated at 37°C for 5 minutes. Next, the exosomes were added with 2 mL PBS containing 0.1% bovine serum albumin to stop staining and then centrifuged at 110 000 *g* for 1 hour at 4°C. The supernatant was removed, and then, the exosomes were supplemented with appropriate medium for suspension. The nuclei of PANC‐1 cells were stained with 4',6‐diamidino‐2‐phenylindole (DAPI). The labelled exosomes were added to cultured PANC‐1 cells to a final concentration of 10 mg/L. Four hours later, a fluorescence microscope was used to observe the uptake of fluorescence‐labelled exosomes by PANC‐1 cells.

### 2.4 Cell treatment, grouping and transfection

In order to observe the effect of M2 macrophage‐derived exosomes on PANC‐1 cells, PANC‐1 cells were grouped as the control group (PANC‐1 cells without any treatment) and the Mp‐Exo group (cells co‐cultured with 200 μg of M2 macrophage‐derived exosomes for 48 hours). In order to observe the effect of M2 macrophage‐derived exosome‐carried lncRNA SBF2‐AS1 on PANC‐1 cells, PANC‐1 cells were assigned into the overexpression (oe)‐negative control (NC) group (PANC‐1 cells transfected with lncRNA SBF2‐AS1 overexpression plasmid NC), the oe‐SBF2‐AS1 group (PANC‐1 cells transfected with lncRNA SBF2‐AS1 overexpression plasmid), the Mp‐Exo‐sh‐NC group (PANC‐1 cells co‐cultured with 200 μg of M2 macrophage‐derived exosomes transfected with lncRNA SBF2‐AS1 interference plasmidNC for 48 hours) and the Mp‐Exo‐sh‐SBF2‐AS1 group (PANC‐1 cells co‐cultured with 200 μg of M2 macrophage‐derived exosomes transfected with lncRNA SBF2‐AS1 interference plasmid for 48 hours).

#### 2.4.1 Cell transfection

When the M2 macrophages and PANC‐1 cells reached 80%‐90% confluence, the M2 macrophages were transfected with lncRNA SBF2‐AS1 interference vectorand its NC while PANC‐1 cells with lncRNA SBF2‐AS1 overexpression plasmid and its NC according to the Lipofectamine 2000 specification (11668‐019, Invitrogen). Exosomes were extracted after transfection. LncRNA SBF2‐AS1 interference vector and its NC, lncRNA SBF2‐AS1 overexpression plasmid and its NC were purchased from GenePharma Ltd. Company.

### 2.5 5‐Ethynyl‐2′‐deoxyuridine (EdU) assay

The cells were inoculated in a 96‐well plate at 4 × 10^3^ cells per well and cultured to 80% confluence, followed by cell proliferation detection by an EdU kit (RiboBio). The cells were incubated in a new EdU medium (100 μL, 50 μm, diluted with a cell culture medium at 1000:1) for 2 hours before two PBS washes (5 minutes once) with the medium removed. Subsequently, the cells were fixed by 50 μL 4% paraformaldehyde, incubated with 50 μL 2 mg/mL glycine and supplemented with 100 μL 0.5% Triton X‐100 penetrant in each well for 10‐min incubation before 5‐min PBS washes. Then, the cells were cultivated with 100 μL 1 × Apollo® staining reaction solution avoiding light for 30 minutes, infiltrated, de‐colorized with methanol, stained with DAPI and captured by a laser confocal scanning microscope (Leica).

### 2.6 Flow cytometry

The cells were detached with 0.25% trypsin after 48‐hours transfection (PYG0107, Boster) and centrifuged twice with the supernatant eliminated. Based on the Annexin‐V‐fluorescein isothiocyanate (FITC) Apoptosis Detection Kit instructions (K201‐100, BioVision), the Annexin‐V‐FITC‐propidium iodide (PI) staining solution was prepared with Annexin‐V‐FITC, PI and hydroxyethyl piperazine ethanesulfonic acid (HEPES) buffer at 1:2:50. The cells (1 × 10^6^) were resuspended in 100 µL of the staining solution, incubated for 15 minutes and added with 15 mL of HEPES buffer. The FITC and PI fluorescence were detected at 515 and 620 nm to detect cell apoptosis.

### 2.7 Scratch test

After 48 hours of transfection, the PC cells were inoculated on 6‐well plates at 5 × 10^5 ^cells/well. When complete cell adherence occurred, a 2‐mm cell scraper was used to scratch in the middle of each well, followed by 24‐h culture. Photographs were taken at the 0 and 24th hours after cell scratching, and the scratch distance was calculated with Image‐Pro plus 6.0.

### 2.8 Transwell assay

The Matrigel‐coated Transwell chamber was preheated to 37°C to detach the transfected cells. The cells were grouped as the above way. The transfected cells were resuspended in serum‐free medium and altered to 1 × 10^5^ cells/mL after counting. RPMI 1640 medium (600 μL) containing 20% FBS was appended to the lower chamber, while the cell suspension (200 mL) was jointed to the upper chamber for 48‐hours culture at 37°C. Then, the cells on the side membrane of the upper chamber were wiped off. After PBS washing, the cells were fastened with 4% paraformaldehyde solution, stained with crystal violet staining solution and observed under a light microscope. Five random high‐power fields were chosen for counting. Three duplicates were set in each group.

### 2.9 RNA‐fluorescence in situ hybridization (FISH) assay

FISH assay was conducted to identify the subcellular localization of lncRNA SBF2‐AS1 in cells. This assay was performed following the requirements of Ribo™ lncRNA FISH Probe Mix (Red, RiboBio Co., Ltd.). To begin with, the cells were seeded on a coverslip on the 24‐well culture plate at 6 × 10^4^ cells/well to achieve about 80% confluence and fastened by 1 mL 4% paraformaldehyde fixation. After proteinase K, glycine and acetamidine reagent treatment, 250 μL prehybridization solution was added for incubation, which was then replaced with lncRNA SBF2‐AS1 hybridization solution containing probe overnight at 42°C. Diluted DAPI (ab104139, 1:100, Abcam) was added for 5‐min cell staining. Finally, the plate was sealed with an anti‐fluorescence quencher, followed by fluorescence microscopy observation (Olympus) and photographing.

### 2.10 Dual‐luciferase reporter gene assay

The wild‐type (WT) and mutant type (MUT) sequences of lncRNA SBF2‐AS1 and XIAP mRNA 3′‐untranslated regions (3′‐UTR) were inserted into the pmiR‐RB‐REPORT™ vector (RiboBio). The correctly sequenced WT and MUT were transfected with mimic‐NC, miR‐122‐5p mimic into 293T cells. After 48‐h transfection, the cells were lysed before 3‐ to 5‐min centrifugation. With the firefly luciferase as a loading control, the relative light unit value of Renilla luciferase divided by that of firefly luciferase was the relative fluorescence value.

### 2.11 RNA immunoprecipitation (RIP) and RNA pull‐down assays

RIP kit (Millipore) was applied for detecting the binding of lncRNA SBF2‐AS1 and Ago2. The antibodies applied in RIP were rabbit anti‐Ago2 (ab186733, 1:50), and rabbit anti‐IgG (ab109489, 1:100, Abcam) was used as NC. The relationship between lncRNA SBF2‐AS1 and miR‐122‐5p was further verified by RNA pull‐down assay.^25^ The binding RNA was purified by TRIzol, and reverse transcription quantitative PCR (RT‐qPCR) was applied for the determination of lncRNA SBF2‐AS1 enrichment.

### 2.12 RT‐qPCR

TRIzol method (Takara) was applied for extracting total RNA from both cells and tissues. On the basis of the reverse transcription kits (K1621, Fermentas), cDNA was obtained by RNA reverse transcription. LncRNA SBF2‐AS1, miR‐122‐5p, XIAP and other genes’ primer sequences were entrusted by Shanghai GeneChem Co., Ltd. (Table 1). Fluorescence quantitative PCR kit (Takara,) was adopted to lncRNA, mRNA and miRNA detection by employing RT‐qPCR (ABI 7500, ABI). U6 was a loading control for miR‐122‐5p and glyceraldehyde phosphate dehydrogenase (GAPDH) for lncRNA SBF2‐AS1, XIAP and other genes. 2^‐ΔΔCt^ method was utilized for the calculation of the relative expression of target genes.

**Table 1:** Primer sequence

Gene	Primer sequence (5′–3′)
SBF2‐AS1	Forward: 5′‐AGACCATGTGGACCTGTCACTG‐3′
	Reverse: 5′‐GTTTGGAGTGGTAGAAATCTGTC‐3′
miR‐122‐5p	Forward: 5′‐GGGTGGAGTGTGACAATGG‐3′
	Reverse: 5′‐CAGTGCGTGTCGTGGAGT‐3′
CD206	Forward: 5′‐CAAGGAAGGTTGGCATTTGT‐3′
	Reverse: 5′‐CCTTTCAGTCCTTTGCAAGC‐3′
CD68	Forward: 5′‐GCTACATGGCGGTGGAGTACAA‐3′
	Reverse: 5′‐ATGATGAGAGGCAGCAAGATGG‐3′
iNOS	Forward: 5′‐ACAGGAGGGGTTAAAGCTGC‐3′
	Reverse: 5′‐TTGTCTCCAAGGGACCAGG‐3′
Arginase	Forward: 5′‐TTGGCAATTGGAAGCATCTCTGGC‐3′
	Reverse: 5′‐TCCACTTGTGGTTGTCAGTGGAGT‐3′
XIAP	Forward: 5′‐GTGACTAGATGTCCACAAGG‐3′
	Reverse: 5′‐GTTGAGGAGTGTCTGGTAAG‐3′
U6	Forward: 5′‐CTCGCTTCGGCAGCACA‐3′
	Reverse: 5′‐AACGCTTCACGAATTTGCGT‐3′
GAPDH	Forward: 5′‐TCCCATCACCATCTTCCA‐3′
	Reverse: 5′‐CATCACGCCACAGTTTTCC‐3′

Abbreviations: GAPDH, glyceraldehyde‐3‐phosphate dehydrogenase; miR‐122‐5p, micro‐122‐15p; XIAP, X‐linked inhibitor of apoptosis protein.

### 2.13 Western blot analysis

The protein concentration was tested based on BCA kit (AR0146, Boster). The extracted proteins were separated with 10% polyacrylamide gel electrophoresis and then transferred onto a polyvinylidene fluoride membrane (P2438, Sigma‐Aldrich), followed by 1‐h 5% bovine serum albumin (10‐L16, Zhongshenglikang) sealing. Then, membranes were mixed with the primary antibodies XIAP (1:1000), CD63 (1:1000), TSG101 (1:1000), CD81 (1:1000) and GRP94 (1:1000) (all from Abcam, Cambridge, MA, USA) overnight at 4°C as well as the corresponding secondary antibody (ab6721, 1:2000, Abcam). Chemiluminescence reagents were utilized for development, with GADPH (ab181602, 1:2000, Abcam) as a loading control. The images were captured, and the grey values of target protein bands were processed by an ImageJ software.

### 2.14 Tumour xenografts in nude mice

Thirty‐two BALB/c female nude mice were obtained from the Experimental Animal Center of the Chinese Academy of Science (4‐6 weeks old, weighing 16‐22 g). Nude mice were randomly grouped as follows (eight rats in each group): the control group (nude mice injected with normal saline), the Mp‐Exo group (nude mice injected with M2 macrophage‐derived exosomes), the Mp‐Exo‐sh‐NC group (nude mice injected with M2 macrophage‐derived exosomes transfected with  lncRNA SBF2‐AS1 interference NC) and the Mp‐Exo‐sh‐SBF2‐AS1 group (nude mice injected with M2 macrophage‐derived exosomes transfected with  lncRNA SBF2‐AS1 interference plasmid). PANC‐1 cells were altered to 1 × 10^6^ cells/100 μL by PBS. Then, 100 μL suspension was injected into the groin of nude mice through caudal vein. The nude mice in the Mp‐Exo group, the Mp‐Exo‐sh‐NC group and the Mp‐Exo‐sh‐SBF2‐AS1 group were injected with 200 μg of exosome suspension every 2 days after modelling with a total of 10 injections. The nude mice in the control group were injected with the same volume of saline as the exosome suspension as a control. Next, the mice were raised in the animal experiment centre. From the 7th day after modelling, the long diameter (*L*), width diameter (*W*) and weight of tumour were measured every 7 days (Volume [*V*] = *W* ^2^ × *L* × 0.52).^26^ The nude mice were killed on the 35th day, and the transplanted tumours were taken out. The largest surface of nude mice’ tumour tissues was removed avoiding the necrotic tissues. Excessive parts were fastened in 4% neutral formaldehyde solution, embedded by paraffin (50 μm apart) and cut into 10 sections (5‐μm) for subsequent experiments.

### 2.15 Statistical analysis

All data were analysed with the SPSS 21.0 software package (IBM Corp.) and consistent with the normal distribution and the homogeneity variance test. The measurement data were depicted as mean ± standard deviation. Comparisons between two groups were made by independent sample *t* test while those among multiple groups by one‐way analysis of variance (ANOVA), after which Tukey's post hoc test was performed. The difference was statistically significant at *P* < .05.

## 3 RESULTS

### 3.1 Isolation and identification of M2 macrophage‐derived exosomes

**Figure 1:**
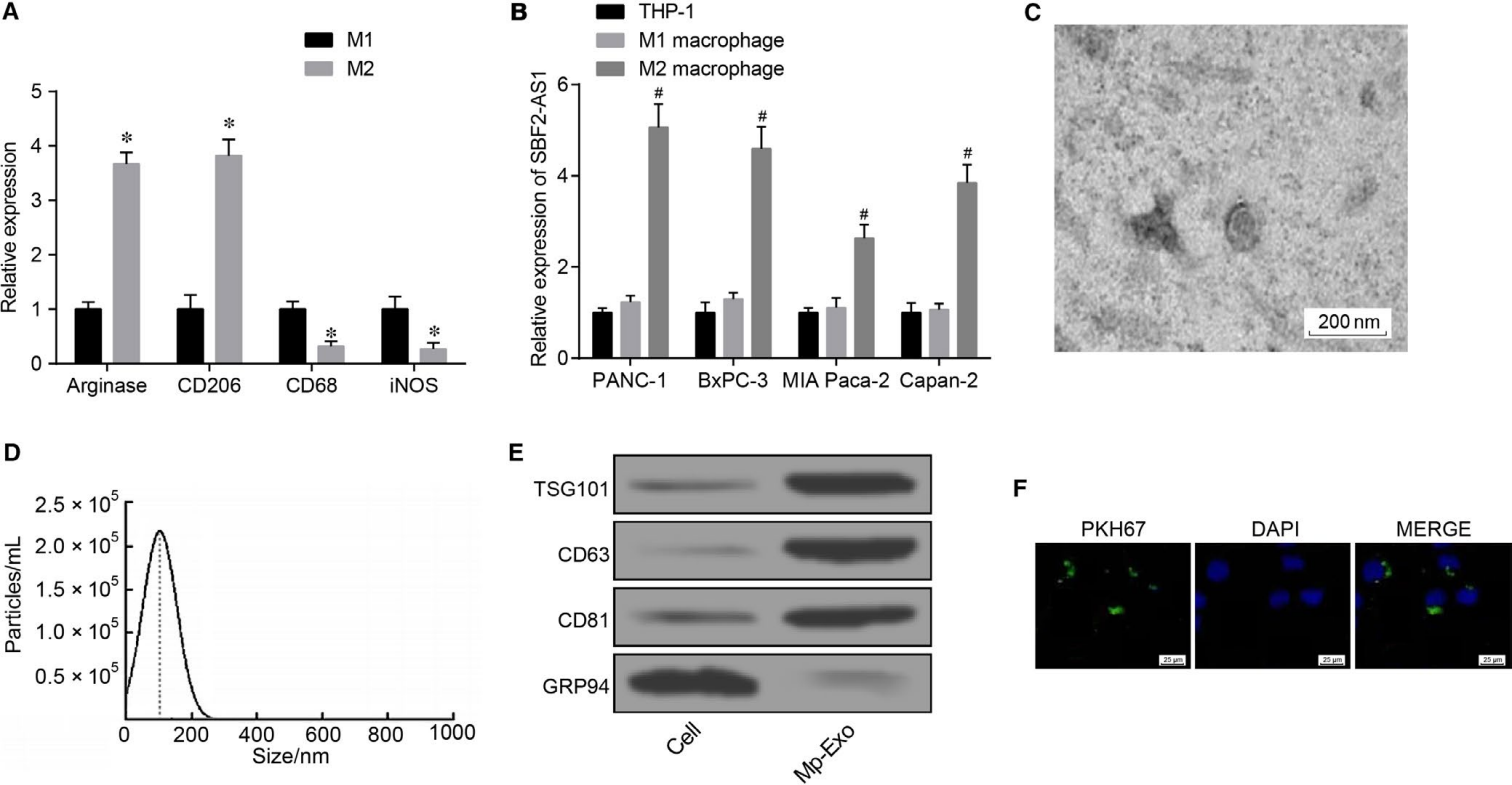
Isolation and identification of M2 macrophage‐derived exosomes. A, RT‐qPCR detection of M1 and M2 macrophage marker gene expression; B, RT‐qPCR detection of lncRNA SBF2‐AS1 expression in each PC cell line; C, observation of exosome morphological characteristics by TEM (scale bar = 200 nm); D, detection of exosome diameter by NanoSight particle‐tracking analysis; E, Western blot analysis of the expression of exosome markers (TSG101, CD63, CD81 and GRP94); F, uptake of PKH‐67‐labelled M2 macrophage exosomes by PANC‐1 cells (scale bar = 25 µm); *, *P* < .05 vs the M1 group; #, *P* < .05 vs the THP‐1 cells; all the above results were measurement data expressed by mean ± standard deviation; statistical analysis was performed by independent sample *t* test and comparison among multiple  groups was analyzed by one‐way ANOVA; the experiment was repeated three times

THP‐1 cells were transformed into M1 macrophage phenotypes after LPS and γ‐IFN treatment, and expressed M1 marker genes (CD68 and inducible nitric oxide synthase). Treated with IL‐4, THP‐1 cells were transformed into M2 macrophages and expressed M2 marker genes (arginase and CD206) (Figure 1A; *P* < .05). LncRNA SBF2‐AS1 expression was detected after co‐culture of PC cell lines and M2 macrophages. The findings illustrated that lncRNA SBF2‐AS1 expression in M2 macrophage co‐cultured with PC cells was substantially up‐regulated vs THP‐1 cells co‐cultured with PC cells (Figure 1B; *P* < .05). PANC‐1 cells with the highest lncRNA SBF2‐AS1 expression were chosen for subsequent assays. TEM findings indicated that exosomes existed in the cell culture supernatant of M2 macrophage, and were solid and dense, possessed with a typical two‐layer membrane, which was disc‐ or cup‐shaped with a mean diameter of 95 nm (Figure 1C,D). Western blot assay was employed to examine the exosome marker proteins (TSG101, CD63, CD81) and non‐exosome marker protein GRP94. TSG101, CD63 and CD81 were expressed, but GRP94 was not expressed by M2 macrophage‐derived exosomes, which further confirmed the success of exosome isolation (Figure 1E).

PKH67‐labelled M2 macrophage exosomes were co‐cultured with PANC‐1 cells for 48 h, and the uptake of exosomes by PANC‐1 cells was observed under an inverted fluorescence microscope. The results showed that PKH67‐labelled exosomes with green fluorescent appeared in the co‐culture group of PANC‐1 and exosomes (Figure 1F).

### 3.2 M2 macrophage‐secreted exosomes induce progression and tumour xenografts of PC cells

**Figure 2:**
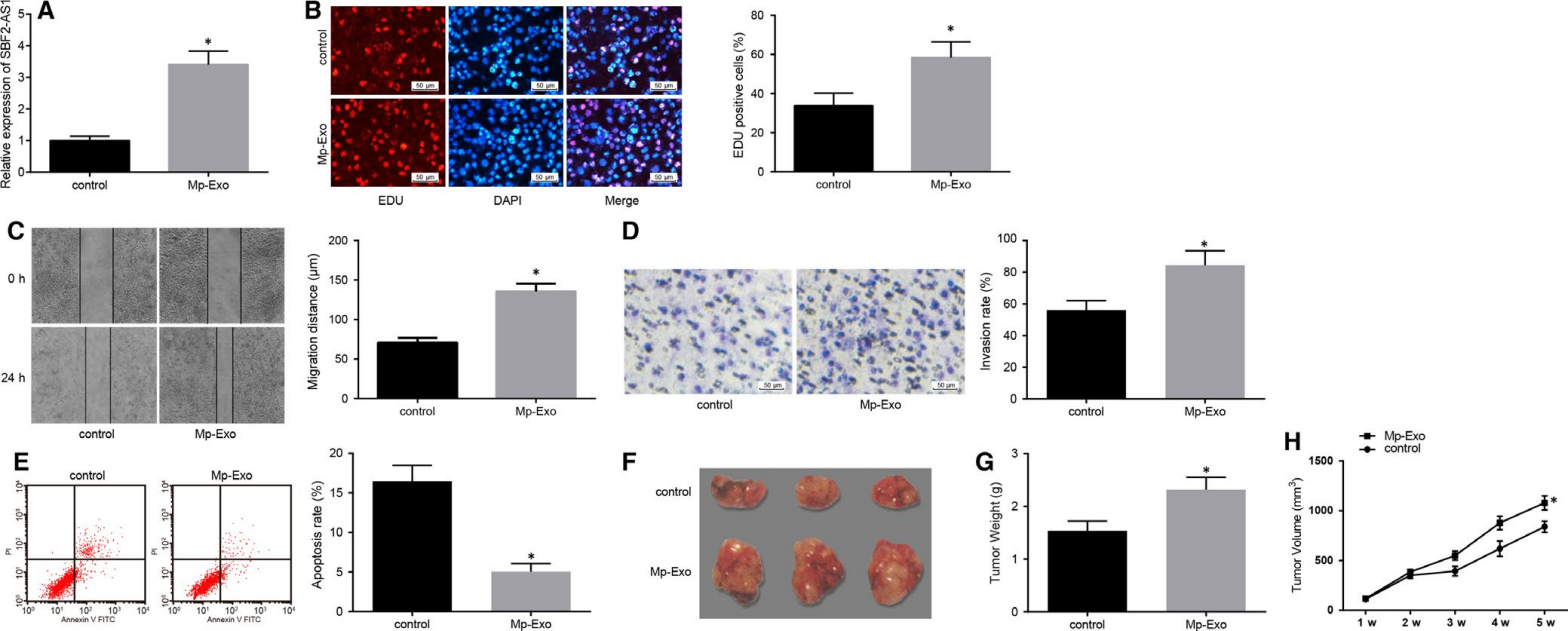
M2 macrophage‐derived exosomes promote proliferation, migration, invasion and tumour xenograft of PC cells and restrict their apoptosis. A, RT‐qPCR detection of lncRNA SBF2‐AS1 expression in PANC‐1 cells after Mp‐Exo treatment; B, EdU detection of changes in PANC‐1 cell proliferation after Mp‐Exo treatment (scale bar = 50 μm); C, detection of changes in PANC‐1 cell migration after treatment with Mp‐Exo by scratch test; D, detection of PANC‐1 cell invasion after Mp‐Exo treatment by Transwell assay (scale bar = 50 μm); E, apoptosis detection of PANC‐1 cells after treatment with Mp‐Exo by flow cytometry; F, representative figures of tumour xenograft in nude mice; G, tumour weight change in each group of nude mice (n = 8); H, tumour volume change in each group of nude mice (n = 8); *, *P* < .05 vs the control group; all the above results were measurement data expressed by mean ± standard deviation, and comparison between two groups was analysed by independent sample *t* test; the cell experiment was repeated three times

After co‐culture of PANC‐1 and M2 macrophage exosomes for 48 h, the expression of lncRNA SBF2‐AS1 detected by RT‐qPCR showed that compared with the control group, the expression of lncRNA SBF2‐AS1 in the Mp‐Exo group was increased (Figure 2A). EdU assay, flow cytometry and Transwell assay mirrored that the ability of proliferation, migration and invasion of PC cells in the Mp‐Exo group rose markedly (Figure 2B‐D), while cell apoptosis ability fell a lot vs the control group (Figure 2E; all *P* < .05). Subcutaneous xenograft mouse models of PC were established using PC cell lines (Figure 2F), and the results suggested an increase in tumour volume and weight of mice in the Mp‐Exo group (Figure 2G,H; *P* < .05). The above findings implied that M2 macrophage‐derived exosomes promoted PC cell progression and tumour xenograft in nude mice.

### 3.3 Overexpressed lncRNA SBF2‐AS1 in M2 macrophage‐secreted exosomes induces progression and tumour xenograft of PC cells

**Figure 3:**
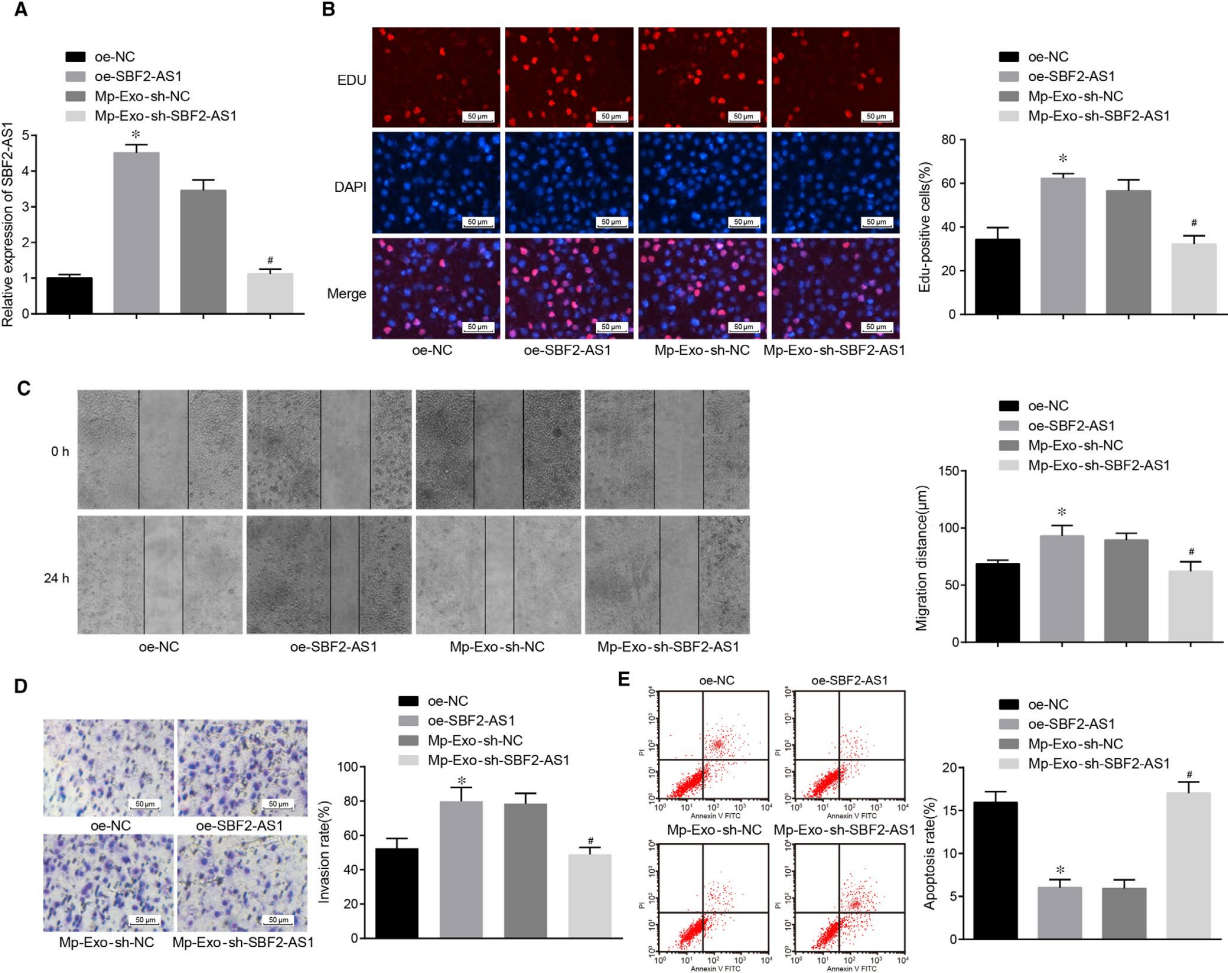
Overexpressed lncRNA SBF2‐AS1 in M2 macrophage‐derived exosomes promote progression of PC cells. A, RT‐qPCR detection of lncRNA SBF2‐AS1 expression in PANC‐1 cells of each group; B, EdU detection of PANC‐1 cell proliferation in each group (scale bar = 50 μm); C, detection of PANC‐1 cell migration in each group by scratch test; D, detection of PANC‐1 cell invasion in each group by Transwell assay (scale bar = 50 μm); E, detection of PANC‐1 cell apoptosis in each group by flow cytometry; *, *P* < .05 vs the oe‐NC group; #, *P* < .05 vs the Mp‐Exo‐sh‐NC group; all the above results were all measurement data expressed as mean ± standard deviation, and comparison among multiple groups was analyzed by one‐way ANOVA; the cell experiment was repeated three times

With the aim to elucidate the function of lncRNA SBF2‐AS1 on PC cells, PANC‐1 cells were transfected with lncRNA SBF2‐AS1 overexpression plasmid and incubated with lncRNA SBF2‐AS1 interference plasmid‐treated M2 macrophage‐derived exosomes  (Figure 3A) (*P* < .05). The findings of EdU assay, flow cytometry and Transwell assay showed that lncRNA SBF2‐AS1 overexpression promoted the PANC‐1 cell proliferation, migration and invasion abilities (Figure 3B‐D; all *P* < .05), and restrained cell apoptosis (Figure 3E; *P* < .05). The above results in the Mp‐Exo‐sh‐SBF2‐AS1 group were opposite to the oe‐SBF2‐AS1 group and the Mp‐Exo group. Namely, lncRNA SBF2‐AS1 and M2 macrophage‐derived exosomes had the accordant impact on PC cells, and depressed lncRNA SBF2‐AS1 in M2 macrophage‐derived exosomes can reverse the effect of M2 macrophage‐derived exosomes on PANC‐1 cells.

### 3.4 LncRNA SBF2‐AS1 represses miR‐122‐5p to up‐regulate XIAP

**Figure 4:**
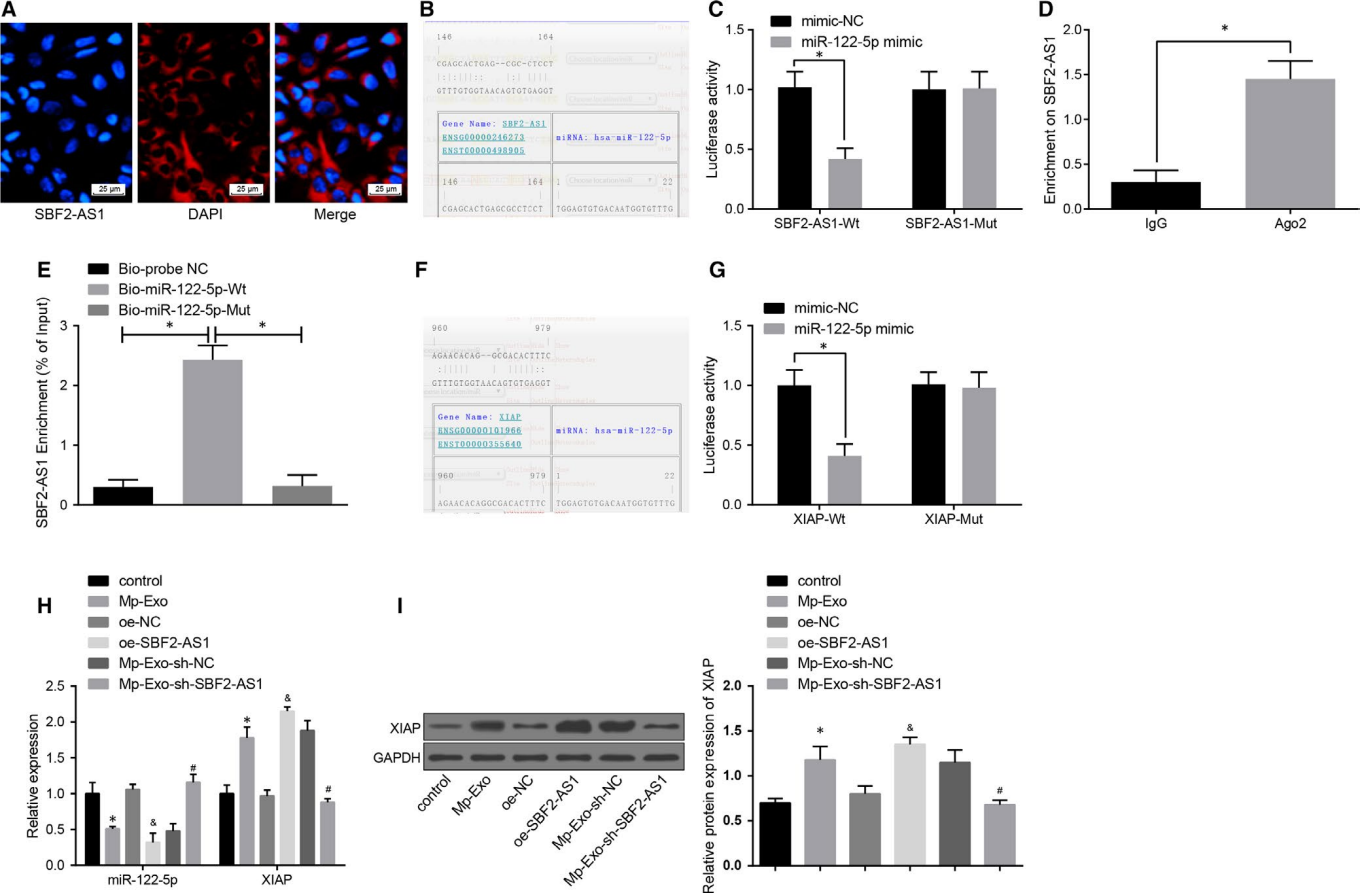
LncRNA SBF2‐AS1 functions as a ceRNA to repress miR‐122‐5p and then up‐regulate XIAP expression. A, Verification of lncRNA SBF2‐AS1 subcellular localization by FISH assay; B, prediction of binding site of lncRNA SBF2‐AS1 and miR‐122‐5p by RNA22 website; C, confirmation of the binding of lncRNA SBF2‐AS1 to miR‐122‐5p by dual‐luciferase reporter gene assay; D, RIP detection of the binding of lncRNA SBF2‐AS1 to Ago2; E, RNA pull‐down detection of the enrichment of miR‐122‐5p to lncRNA SBF2‐AS1; F, prediction of the binding site between miR‐122‐5p and XIAP by RNA22 site; G, verification of the binding of miR‐122‐5p to XIAP by dual‐luciferase reporter gene assay; H, expression of miR‐122‐5p and XIAP in PC cells detected by RT‐qPCR; I, expression of XIAP in PC cells detected by Western blot analysis; *, *P* < .05 vs the control group; #, *P* < .05 vs the oe‐NC group; &, *P* < .05 vs the Mp‐Exo‐sh‐NC group; the data in the figure were all measurement data expressed as mean ± standard deviation; comparison between two groups was analysed by independent sample *t* test, and comparison among multiple groups was analysed by one‐way ANOVA; the experiment was repeated three times

RNA‐FISH results showed that lncRNA SBF2‐AS1 was mainly functioned in the cytoplasm (Figure 4A). Through the RNA22 website (https://cm.jefferson.edu/rna22/Precomputed/), it was predicted that lncRNA SBF2‐AS1 could bind to miR‐122‐5p (Figure 4B) and further verified by dual‐luciferase reporter gene assay with the corresponding findings revealing that the luciferase activity of cells cotransfected with WT‐SBF2‐AS1 and miR‐122‐5p mimic descended greatly (*P* < .05), while no palpable difference was seen in the luciferase activity of cells cotransfected with MUT‐SBF2‐AS1 and miR‐122‐5p mimic (*P* > .05), indicating that miR‐122‐5p may specifically bind to lncRNA SBF2‐AS1 (Figure 4C). RIP assay showed that the lncRNA SBF2‐AS1’s specific sponge of Ago2 ascended markedly vs the IgG group (*P* < .05; Figure 4D). RNA pull‐down assay verified that lncRNA SBF2‐AS1 enrichment in the bio‐miR‐122‐5p‐WT group increased clearly (*P* < .05), while no marked difference was seen in the same parameter in the bio‐miR‐122‐5p‐MUT group vs the bio‐probe NC group (*P* > .05; Figure 4E), indicating lncRNA SBF2‐AS1 can function as a sponge to miR‐122‐5p, thereby affecting miR‐122‐5p expression. At the same time, we also predicted the target gene of miR‐122‐5p in the RNA22 website and found that a targeted binding site existed between miR‐122‐5p and XIAP (Figure 4F). Dual‐luciferase reporter gene assay also revealed that (Figure 4G) miR‐122‐5p specifically bound to XIAP, and XIAP was the target gene of miR‐122‐5p. To further verify our inference, we adopted RT‐qPCR and Western blot assay to determine miR‐122‐5p and XIAP expression in PANC‐1 cells, and the results illustrated that (Figure 4H,I) miR‐122‐5p fell substantially, while XIAP expression grew greatly in the Mp‐Exo group and the oe‐SBF2‐AS1 group vs the control group and the oe‐NC group (all *P* < .05). Relative to the Mp‐Exo‐sh‐NC group, the expression of XIAP reduced and the expression of miR‐122‐5p increased in the Mp‐Exo‐sh‐SBF2‐AS1 group (both *P* < .05). Therefore, it was speculated that lncRNA SBF2‐AS1 repressed miR‐122‐5p, thereby up‐regulating XIAP.

### 3.5 Constrained lncRNA SBF2‐AS1 in M2 macrophage‐secreted exosomes restrains the tumorigenic ability of PANC‐1 cells

**Figure 5:**
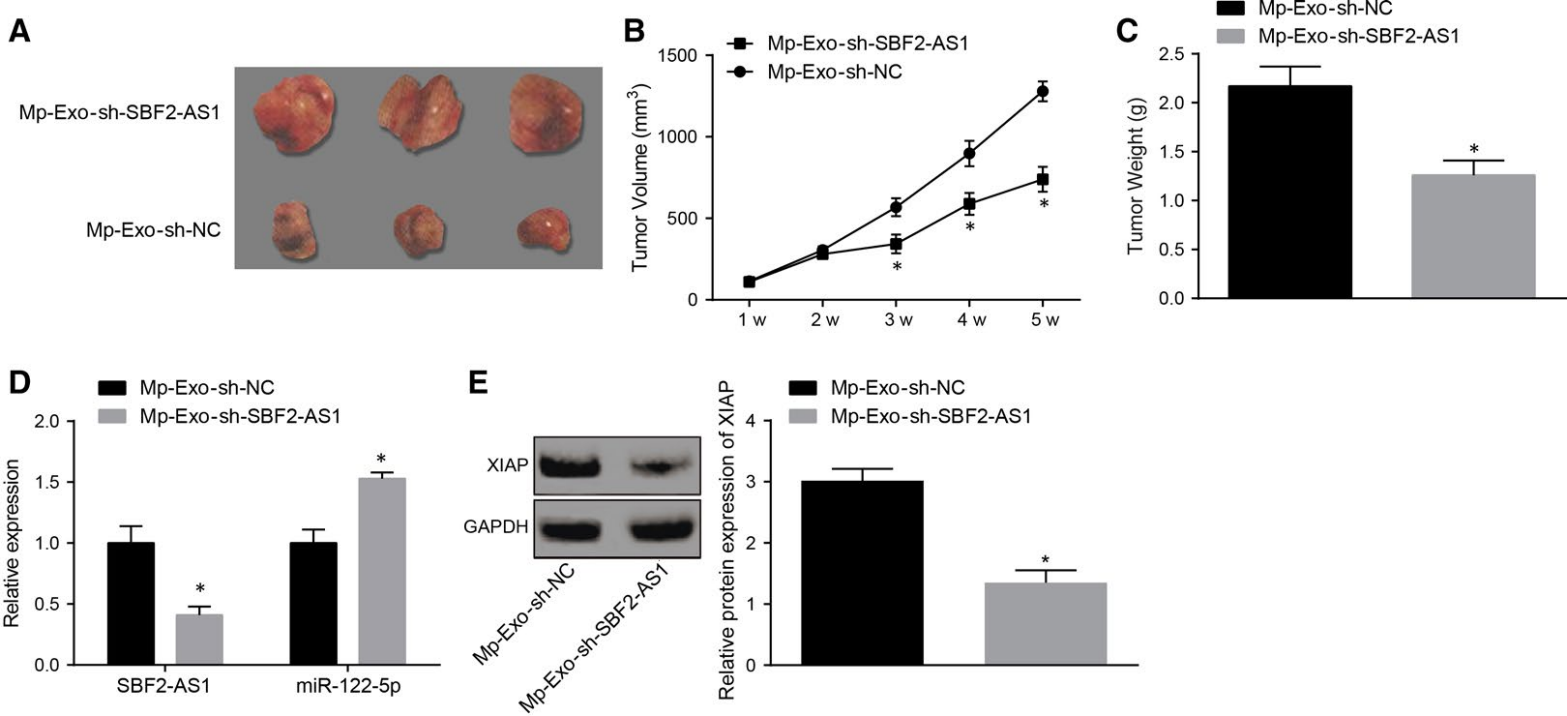
Constrained lncRNA SBF2‐AS1 in M2 macrophage‐derived exosomes restrains the tumorigenic ability of PANC‐1 cells. A, Representative figures of tumour xenograft in nude mice; B, tumour volume change in nude mice in each group (n = 8); C, tumour weight change in nude mice in each group (n = 8); D, RT‐qPCR detection of lncRNA SBF2‐AS1 and miR‐122‐5p expression in PC tissues; E, Western blot analysis detection of XIAP expression in PC tissues; *, *P* < .05 vs the Mp‐Exo‐sh‐NC group; the data in the figure were all measurement data expressed as mean ± standard deviation, and comparison between two groups was analysed by independent sample *t* test; the experiment was repeated three times

Subcutaneous xenograft mouse models of PC were established in the same way as above, and exosomes in the M2 macrophages transfected with sh‐NC or sh‐SBF2‐AS1 were extracted and injected into the nude mice through caudal vein. The subcutaneous tumour weight and volume of mice in the Mp‐Exo‐sh‐SBF2‐AS1 group dropped obviously vs the Mp‐Exo‐sh‐NC group (Figure 5A‐C; *P* < .05), lncRNA SBF2‐AS1 expression in subcutaneous PC tissues was substantially suppressed, and miR‐122‐5p expression was elevated (Figure 5D,E; *P* < .05). The obtained results from Western blot analysis mirrored that XIAP expression in Mp‐Exo‐sh‐SBF2‐AS1 group was down‐regulated to a large extent vs the Mp‐Exo‐sh‐NC group (Figure 5F; *P* < .05). It was indicated that constrained lncRNA SBF2‐AS1 in M2 macrophage‐secreted exosomes restrained the tumorigenic ability of PANC‐1 cells.

## 4 DISCUSSION

Pancreatic cancer has been reported to be the fourth most common cancer to lead to death, and the highest 5‐year survival rate is only 31.5% for patients after treatment.^27^ Though the cause of PC is complicated and multifaceted, family history and smoking are considered as the main factors.^28^ Recently, M2 macrophages have been discovered to have something to do with gastric and breast cancer.^29^ Nevertheless, the role of M2 macrophage exosomes in PC remains to be elucidated. Hence, this study is meant to discuss how M2 macrophages affect malignant phenotypes of PC through lncRNA SBF2‐AS1/miR‐122‐5p/XIAP axis. Collectively, our study revealed that silencing of lncRNA SBF2‐AS1 in M2 macrophage exosomes increased miR‐122‐5p expression to repress XIAP expression, which further depressed PC progression.

Through a number of assays, we found that M2 macrophage exosomes promoted progression and tumour xenograft of PC cells and restrict their apoptosis. In consistent with our study, a previous study has reported that exosomes derived from macrophages have the ability to modulate drug resistance in pancreatic adenocarcinoma.^29^ It has been indicated that in breast cancer, exosomes are capable of mediating oncogenic information among cells systematically and contribute greatly to cancer development via horizontal transfer of a variety of bioactive molecules.^30^ Also, evidence has illustrated that exosomes can promote angiogenesis and tumour invasion by activating matrix metalloproteinase in fibroblasts; meanwhile, create environments beneficial to tumour progression by regulating the activity of immune cells.^31^ All these findings are in full support of our result that M2 macrophage exosomes facilitate progression of PC cells.

Moreover, we found that overexpressed lncRNA SBF2‐AS1 in M2 macrophage exosomes promoted cell progression and tumour xenograft of PC, and lncRNA SBF2‐AS1 silencing in M2 macrophage‐secreted exosomes restrained the tumorigenic ability of PC cells. As we know from a previous study, it is concluded that lncRNA SBF2‐AS1 down‐regulation restrains TWF1 expression by competitively binding with miR‐142‐3p to facilitate gemcitabine resistance in PC.^10^ In accordance with our study, there is evidence proved that lncRNA SBF2‐AS1 is elevated in non–small‐cell lung cancer (NSCLC) and that overexpressed lncRNA SBF2‐AS1 boosts NSCLC proliferation.^32^ Excessive lncRNA SBF2‐AS1 is also found in hepatocellular carcinoma (HCC), and further experiments have verified that lncRNA SBF2‐AS1 depletion can repress HCC cell proliferation and slow HCC tumour growth in vivo.^33^ Moreover, a similar study has revealed that lncRNA SBF2‐AS1 is highly enhanced in colorectal cancer (CRC) and lncRNA SBF2‐AS1 restriction exerts negative effects on the CRC development.^34^ It has also been suggested that tumour‐derived exosomal lncRNA Sox2ot is able to alter the phenotype of pancreatic ductal adenocarcinoma cells by impacting epithelial‐mesenchymal transition and stemness.^35^

Another critical finding of our study was that lncRNA SBF2‐AS1 served as a sponge to repress miR‐122‐5p and up‐regulate XIAP expression. Similar to our study, evidence has shown that lncRNA SBF2‐AS1 acts as a miR‐361‐5p sponge to promote FOXM1 expression and boost cervical cancer development consequently.^12^ It is suggested that lncRNA SBF2‐AS1 is able to sponge miR‐338‐3p, which exerts further effects on glioblastoma development.^36^ Recently, Li *et al* have testified that miR‐23a up‐regulation can depress XIAP expression to a large degree, which can function in trophoblast cell apoptosis.^37^ Also, there has been study indicating that a binding site exists between miR‐381 and XIAP, and that increased miR‐381 expression can negatively act on XIAP expression in oesophageal squamous cell carcinoma.^38^

All in all, our study revealed that knock‐down of lncRNA SBF2‐AS1 in M2 macrophage exosomes promoted miR‐122‐5p expression and then declined XIAP expression, thereby constraining PC development (Figure S1). This study provides new clues for the function of lncRNA SBF2‐AS1/miR‐122‐5p/XIAP axis in PC progression and more significantly, a new direction for PC treatment, which is of great importance to human beings. Nevertheless, more studies are necessary to further elaborate the relevant mechanisms of M2 macrophage‐derived exosomes on PC development.

## CONFLICT OF INTEREST

The authors declare that they have no conflicts of interest.

Consent for publication: Not applicable.

Availability of data and material: Not applicable.

## AUTHOR CONTRIBUTIONS

Guarantor of integrity of the entire study: Zi Yin and Yu Zhou. Study design: Tingting Ma and Sheng Chen. Experimental studies: Ning Shi and Yiping Zou. Manuscript editing: Baohua Hou and Chuanzhao Zhang.

## ETHICAL STATEMENT

Animals were treated humanely, and the protocol was approved by the Institutional Animal Care and Use Committee of Guangdong Provincial People's Hospital, Guangdong Academy of Medical Sciences.

## Supporting information

image S1

Figure S1
