# Supplementary figures and images for: Down‐regulated lncRNA SBF2‐AS1 in M2 macrophage‐derived exosomes elevates miR‐122‐5p to restrict XIAP, thereby limiting pancreatic cancer development
Source: J Cell Mol Med. 2020 Apr 16;24(9):5028–38. doi: 10.1111/jcmm.15125 (PMC7205800; doi:10.1111/jcmm.15125)

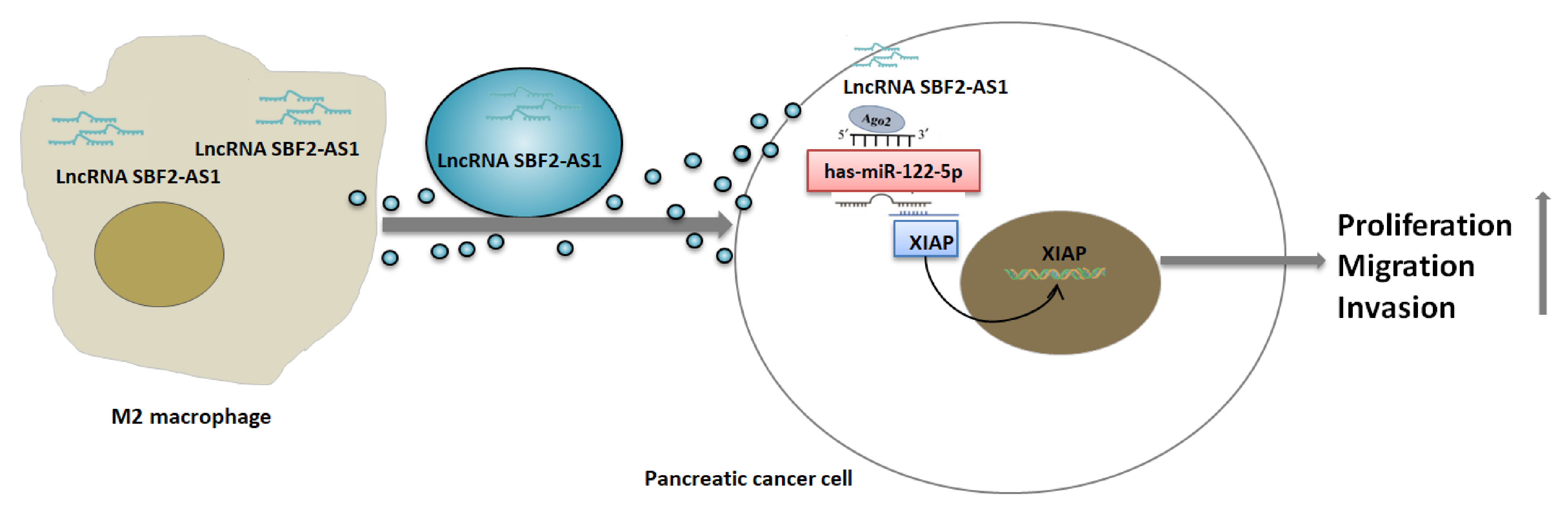

Supplement: Supplementary file 1 — image S1 [file JCMM-24-5028-s001.jpg]
