# Supplementary material for: Down‐regulated lncRNA SBF2‐AS1 in M2 macrophage‐derived exosomes elevates miR‐122‐5p to restrict XIAP, thereby limiting pancreatic cancer development
Source: J Cell Mol Med. 2020 Apr 16;24(9):5028–38. doi: 10.1111/jcmm.15125 (PMC7205800; doi:10.1111/jcmm.15125)
Supplement: Supplementary file 2 — Figure S1 [file JCMM-24-5028-s002.docx]

**Figure S1** Schematic representation of macrophage-derived exosomal lncRNA SBF2-AS1 in PC cells and the involvement of lncRNA SBF2-AS1/miR-122-5p/XIAP axis. M2 macrophages deliver lncRNA SBF2-AS1 through exosomes in PC cells and sponge miR-122-5p, thereby up-regulating XIAP expression and ultimately accelerating the development of PC *in vitro* and *in vivo*.
